# Haemolysis and renal effects across pulsed field ablation systems in atrial fibrillation

**DOI:** 10.1093/europace/euag190

**Published:** 2026-07-30

**Authors:** Matteo Rocchetti, David Schaack, Aldo Marrese, Andrea Urbani, Joseph Kheir, Soroosh Najafi, Melanie Gunawardene, Alexandra Marx, Julia Lurz, Boris Schmidt, Julian Chun, Lukas Urbanek

**Affiliations:** Cardioangiologisches Centrum Bethanien, Agaplesion Markus-Krankenhaus, Wilhelm-Epstein-Str. 4, Frankfurt, Germany; Cardioangiologisches Centrum Bethanien, Agaplesion Markus-Krankenhaus, Wilhelm-Epstein-Str. 4, Frankfurt, Germany; Cardioangiologisches Centrum Bethanien, Agaplesion Markus-Krankenhaus, Wilhelm-Epstein-Str. 4, Frankfurt, Germany; Cardioangiologisches Centrum Bethanien, Agaplesion Markus-Krankenhaus, Wilhelm-Epstein-Str. 4, Frankfurt, Germany; Cardioangiologisches Centrum Bethanien, Agaplesion Markus-Krankenhaus, Wilhelm-Epstein-Str. 4, Frankfurt, Germany; Cardioangiologisches Centrum Bethanien, Agaplesion Markus-Krankenhaus, Wilhelm-Epstein-Str. 4, Frankfurt, Germany; Cardioangiologisches Centrum Bethanien, Agaplesion Markus-Krankenhaus, Wilhelm-Epstein-Str. 4, Frankfurt, Germany; Centre for Heart Rhythm Disorders, University of Adelaide and Royal Adelaide Hospital, Adelaide, SA, Australia; Department of Cardiology, University Hospital Giessen, Giessen, Germany; Cardioangiologisches Centrum Bethanien, Agaplesion Markus-Krankenhaus, Wilhelm-Epstein-Str. 4, Frankfurt, Germany; Cardioangiologisches Centrum Bethanien, Agaplesion Markus-Krankenhaus, Wilhelm-Epstein-Str. 4, Frankfurt, Germany; Department of Cardiology, University Medical Center Mainz, Mainz, Germany; Cardioangiologisches Centrum Bethanien, Agaplesion Markus-Krankenhaus, Wilhelm-Epstein-Str. 4, Frankfurt, Germany; Medizinische Klinik 3-Klinik für Kardiologie, Universitätsklinikum Frankfurt, Frankfurt, Germany; Cardioangiologisches Centrum Bethanien, Agaplesion Markus-Krankenhaus, Wilhelm-Epstein-Str. 4, Frankfurt, Germany; Klinik für Rhythmologie, Universitätsklinikum Schleswig-Holstein der Universität zu LüBeck, Lübeck, Germany; Cardioangiologisches Centrum Bethanien, Agaplesion Markus-Krankenhaus, Wilhelm-Epstein-Str. 4, Frankfurt, Germany; Department of Cardiology, University Medical Center Mainz, Mainz, Germany

**Keywords:** Atrial Fibrillation, Pulsed Field Ablation, Haemolysis

## Abstract

**Aims:**

Pulsed field ablation (PFA) is a non-thermal energy modality for ablation of atrial fibrillation (AF). Although PFA has demonstrated an excellent overall safety profile, increasing evidence suggests an association with intravascular haemolysis, and acute kidney injury (AKI). However, the extent of haemolysis across different PFA catheters remains poorly characterized and warrants further investigation.

**Methods and results:**

We prospectively enrolled 239 patients undergoing AF ablation using the pentaspline (*n* = 107), balloon-in-basket (*n* = 71), or lattice-tip (*n* = 61) catheter. Laboratory markers of haemolysis, myocardial injury, renal function and haemoglobin were collected pre- and 24h post-procedure. Significant haptoglobin depletion was observed exclusively with pentaspline, [pre 99.8 mg/dL (70.2–122) vs. post 55.7 mg/dL (23.2–91.8); *P* < 0.001] Reductions with the lattice-tip (*P* = 0.074) and the balloon-in-basket (*P* = 0.201) catheter did not reach statistical significance. AKI was only observed with the pentaspline catheter (pentaspline: 4.9%; balloon-in-basket: 0.0%; lattice tip: 0.0%; *P* = 0.020). Myocardial markers increased across all groups, but significantly higher in pentaspline and balloon-in-basket (*P* = n.s.) vs. lattice-tip (*P* < 0.001 vs. pentaspline, *P* < 0.001 vs. balloon in basket).

**Conclusion:**

In this large cohort, contemporary PFA systems appear safe but catheter design influences the extent of haemolysis, AKI, and myocardial injury. The pentaspline catheter was associated with the greatest haemolytic effect and higher transient AKI, without clinically relevant reductions in haemoglobin or persistent renal impairment. Higher levels of myocardial injury in the pentaspline and balloon-in-basket groups correspond to the larger lesion footprints of these devices.

## Graphical Abstract

**Graphical Abstract:**
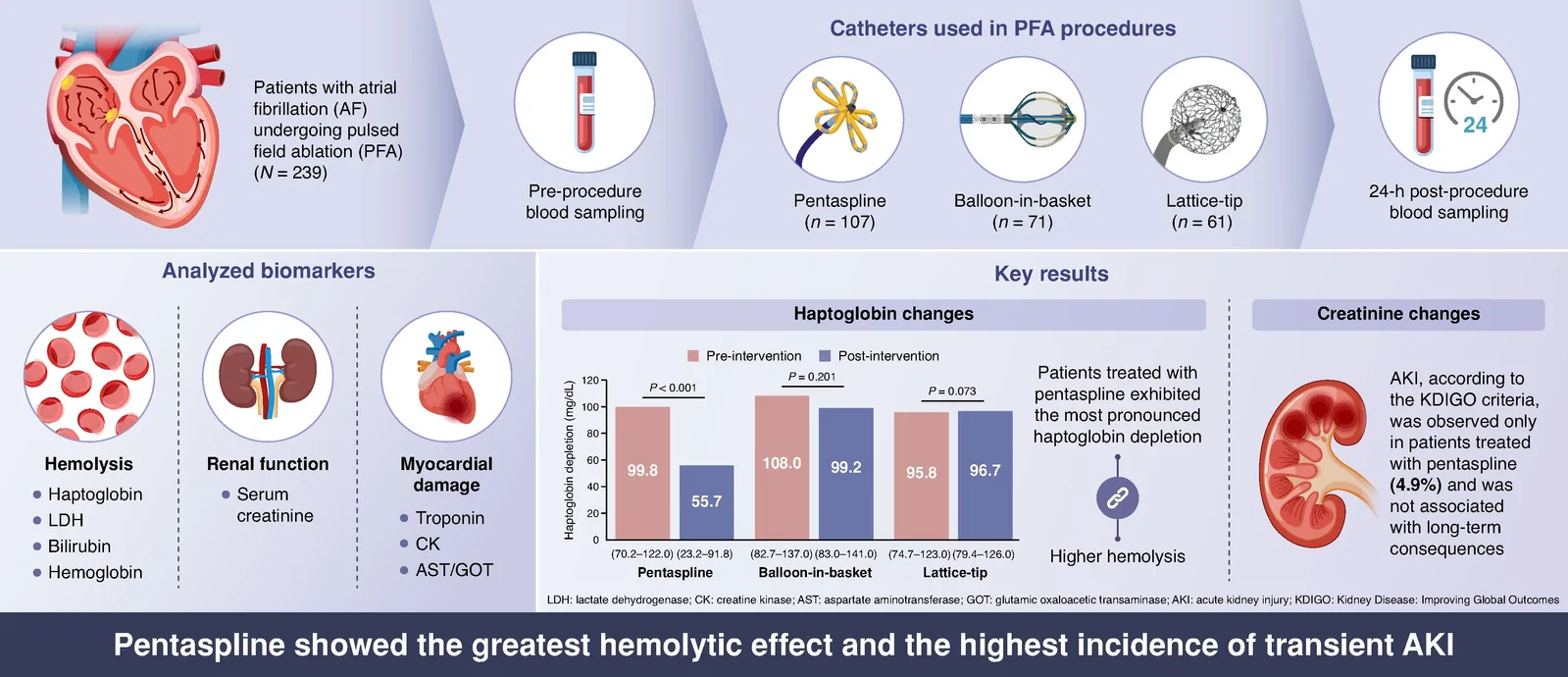

What’s new?

- The pentaspline catheter induced significantly higher haemolysis compared with other PFA platforms, marked by pronounced haptoglobin depletion.
- Haemolysis driven changes led to transient increase in serum creatinine and AKI exclusively in the pentaspline group followed by stable or declining creatinine levels on repeat testing within 48 h.
- Large footprint designs (pentaspline and balloon-in-basket) caused greater myocardial injury than lattice-tip systems.
- Despite similar footprints, the balloon-in-basket catheter caused substantially less haemolysis, suggesting that catheter architecture and contact-sensing mitigate off-target blood-pool energy exposure.

## Introduction

Over the past decade, substantial evidence and clinical experience have accumulated in the field of atrial fibrillation (AF) ablation, leading to continuous refinement of patient selection criteria and procedural indications. This evolution is reflected in the latest joint consensus document issued by the Heart Rhythm Society (HRS), European Heart Rhythm Association (EHRA), Asia Pacific Heart Rhythm Society (APHRS), and Latin American Heart Rhythm Society (LAHRS), which highlights the growing range of catheter-based technologies and strategies currently available for the treatment of AF.^1^

Pulsed field ablation (PFA) has rapidly emerged as a transformative technology for AF ablation, leveraging high-voltage, microsecond-scale electric fields to induce irreversible electroporation and achieve myocardial cell death.^2^ Unlike thermal energy sources, PFA is considered relatively tissue-selective and has demonstrated a favourable safety profile compared with thermal energy, as surrounding tissues such as the oesophagus and the phrenic nerve are largely spared.^3–5^ Taken together, these considerations regarding the benefits and limitations of PFA have contributed to its rapid adoption in clinical practice and support its emergence as a leading ablation technology for the treatment of AF.^6^

Several PFA catheter platforms have been developed and adopted, including the pentaspline catheter,^7^ the balloon-in-basket catheter^8^ and the lattice-tip catheter^9^ integrated with a mapping platform. Early clinical trials and real-world registries have consistently shown high acute efficacy and procedural safety across these technologies, supporting the rapid clinical uptake of PFA.^10,11^

Despite its favourable safety profile, novel complications atypical of thermal energy, notably vasospasm and acute kidney injury (AKI), have emerged. The incidence of AKI has been shown to be attributed to haemolysis, characterized as a reproducible, energy-dependent biological phenomenon.^12^

Haemolysis leads to the release of several biochemical markers, including LDH and unconjugated (indirect) bilirubin, as well as the accumulation of circulating free haemoglobin. Haptoglobin binds free haemoglobin and is therefore reduced in the presence of intravascular haemolysis, making it a sensitive marker of red blood cell destruction. The accumulation of haemoglobin degradation products may induce nephron injury, particularly at the tubular level, potentially resulting in elevations in serum creatinine and, in severe cases, acute renal failure.^13^ In addition, PFA induces selective myocardial cell death, which is reflected by post-procedural increases in biomarkers of myocardial injury such as troponin, creatine kinase (CK), and glutamate-oxaloacetate transaminase (GOT)/aspartate transferase (AST).

Accordingly, multiple studies have reported post-procedural haemolysis, characterized by reductions in haptoglobin and increases in free haemoglobin, lactate dehydrogenase, and bilirubin and consequent creatinine increase. When compared with thermal energy (radiofrequency ablation and cryoballoon ablation), the existing literature consistently suggests that haemolysis is more frequently associated with PFA.^14–17^ The extent of haemolysis appears to be dose-dependent, correlating with the number of PFA applications largely explored for the pentaspline system.^15,16^ Additionally, evidence suggests that the degree of tissue contact represents a critical variable that may further aggravate haemolytic activity in PFA.^18^

Whether the extent of haemolysis is related to the waveform parameters and catheter design is poorly understood and comparable data between different PFA systems is lacking.^14^

In this study, we sought to evaluate haemolysis following AF ablation using PFA, with a specific focus on comparing three widely used catheter platforms: the pentaspline, the lattice-tip and the balloon-in-basket catheters. By integrating laboratory markers of haemolysis with renal and myocardial injury parameters, this work aims to provide a more comprehensive and device-specific assessment of contemporary PFA technologies.

## Methods

### Patient population

We prospectively collected data from patients undergoing PFA-based ablation at our institution between May and December 2025. Patients were treated using one of the three following PFA systems: pentaspline PFA (Farawave™, Boston Scientific, USA), lattice-tip PFA/RF (Sphere-9™, Medtronic, USA), or balloon-in-basket PFA (Volt™, Abbott, USA). Patient selection varied according to the PFA system used. First-time pulmonary vein isolation (PVI) patients were treated with either the pentaspline or the balloon-in basket PFA system according to operator preference and device availability. In contrast, the lattice-tip catheter was mostly used in patients undergoing repeat ablation procedures. Patients that underwent ablation beyond PVI in the balloon-in-basket and the pentaspline group were excluded from the analysis.

### Procedures

All procedures were performed with the patient under deep sedation. Venous groin puncture was performed using ultrasound. Two (for pentaspline or balloon-in-basket) or three (lattice-tip) sheaths were subsequently placed in the right femoral vein and a decapolar diagnostic catheter was placed in the coronary sinus. Access to the left atrium was established via transseptal puncture. Pulmonary vein angiography was performed to provide a true anatomical reference of the left atrium. Procedures were subsequently continued in accordance with the device-specific protocols outlined below.

### Pentaspline procedure

The transseptal sheath was exchanged for the 13.8F Faradrive^TM^ sheath (Boston Scientific^TM^) and subsequently the pentaspline Farawave^TM^ (Boston Scientific^TM^) ablation catheter was introduced. The ablation workflow followed the previously described ‘olive strategy’^19^: a total of 10 standard applications were applied per vein, consisting of four ‘flower,’ four ‘basket,’ and two ‘olive’ shapes. Additional atrial applications were performed at the operator's discretion. Visual fluoroscopy and signal assessment were used to confirm contact, as the catheter did not support contact-force measurement.

### Balloon-in-basket procedure

The transseptal sheath was exchanged for the 13F Agilis^TM^ NxT Dual-Reach sheath (Abbott^TM^) and subsequently the balloon-in-basket Volt^TM^ (Abbott^TM^) ablation catheter was introduced. The ablation followed the ‘pear strategy’, consisting of six applications per vein. Initially, four applications were delivered with the balloon fully inflated (10 mL); the balloon was rotated between applications to ensure circumferential coverage of the inter-electrode gaps. The final two applications were performed with the balloon partially inflated (7 mL) and advanced slightly into the vein to achieve a ‘pear-shaped’ configuration. Tissue contact was confirmed via occlusion angiography or the LivePoint™ (Abbott^TM^) display. For the right pulmonary veins, energy delivery was reduced to the ‘low energy’ setting if phrenic nerve capture was observed.

### Lattice-tip procedure

Following verification of durable PVI, a 3D electroanatomical map was reconstructed using the Affera™ mapping system and Sphere-9™ catheter (Medtronic^TM^).

Electroanatomical mapping was performed during atrial pacing in patients presenting in sinus rhythm and during the clinical tachycardia when present. Following mapping, substrate characterization was based on bipolar voltage measurements provided by the mapping system, using a predefined voltage range of 0.1–0.5 mV and supported by voltage colour maps.

Areas exhibiting low-voltage, heterogeneous, or abnormal electrogram characteristics were considered diseased atrial substrate. When such regions were identified, substrate homogenisation was performed by means of linear lesion sets, including an anterior line, roof line, mitral isthmus line, or posterior wall isolation, according to the anatomical distribution of the substrate.

In patients presenting with atrial tachycardia, activation mapping was used to identify the most likely re-entrant circuit, and the corresponding linear ablation strategy was performed with the aim of tachycardia termination.

Subsequent PFA or RF applications were delivered at the physician's discretion according to the manufacturer’s recommendation. Tissue contact was evaluated via impedance monitoring and confirmed by the optimal lesion efficacy indicators provided by the Affera™ system.

### Statistical analysis

Patients´ baseline characteristics were systematically recorded before the procedure. Baseline continuous variables were compared among groups using the Kruskal–Wallis test, while categorical variables were expressed as counts and percentages and compared using the chi-square test or Fisher’s exact test, as appropriate.

Pre- and post-procedural blood samples were obtained at the beginning of the procedure from the right femoral vein and on the morning of the first post-procedural day. The collected samples primarily included markers of haemolysis, namely haemoglobin, total and indirect bilirubin (mathematically derived), lactate dehydrogenase (LDH), and haptoglobin, together with serum creatinine as the main marker of renal function. In addition, myocardial injury markers, including AST/GOT, creatine kinase (CK), and high-sensitivity troponin T (hs-TnT), were also assessed. Continuous variables were tested for normality using the Shapiro–Wilk test and visual inspection of histograms and Q–Q plots. Continuous variables with non-normal distribution were expressed as median and interquartile range (IQR) and analyzed using non-parametric tests.

The aim of the study was to compare the above mentioned parameters among catheters.

For each continuous parameter, the pre-post difference (Δ) was calculated as the post-procedural value minus the baseline value. Within-group comparisons were performed using the Wilcoxon signed-rank test. Comparisons of Δ values among the three catheter groups were conducted using the Kruskal–Wallis test, followed by post-hoc pairwise comparisons with Dunn’s test and Bonferroni correction when appropriate.

Patients were classified according to the Kidney Disease: Improving Global Outcomes (KDIGO) criteria. An increase in serum creatinine of ≥0.3 mg/dL or 1.5–1.9 times the baseline value was classified as AKI stage 1. An increase of 2.0–2.9 times the baseline value was classified as AKI stage 2. AKI stage 3 was defined as an increase in serum creatinine to ≥3 times baseline, an absolute serum creatinine level ≥4.0 mg/dL, or the initiation of renal replacement therapy.

.^20^ Additional blood sampling after 48 h was performed in patients who developed AKI according to the KDIGO criteria. The incidence of AKI was calculated for each catheter group, and a Fisher exact test was used to compare AKI incidence among catheter types.

Data on total fluid administration were collected for all procedures. For patients treated with the lattice-tip catheter, the recorded fluid volume was adjusted to account for catheter irrigation. Irrigation volume was estimated based on left atrial dwell time and energy delivery mode, assuming flow rates of 4 mL/min during mapping and catheter manipulation, 15 mL/min during PFA delivery, and 30 mL/min during RF delivery. Energy delivery duration was calculated from the number of applications, assuming 4 s per PFA application and 6 s per RF application. Comparisons of fluid administration values among the three catheter groups were conducted using the Kruskal–Wallis test, followed by post-hoc pairwise comparisons with Dunn’s test and Bonferroni correction when appropriate.

All statistical tests were two-tailed, and a *P*-value <0.05 was considered statistically significant. Statistical analyses were performed using SPSS software (IBM Corp., Armonk, NY, USA).

## Results

### Baseline characteristics

A total of 271 consecutive patients undergoing PFA were initially considered for biomarker assessment. To ensure a more homogeneous comparison, only patients undergoing PVI were included in the pentaspline and balloon-in-basket groups. This yielded a final study cohort of 239 patients. Of these, 107 (44.8%) were treated with the pentaspline catheter, 61 (25.5%) with the lattice-tip catheter, and 71 (29.7%) with the balloon-in-basket catheter.

Most baseline characteristics were comparable between groups (*Table 1*). The baseline arrhythmia profile showed significant differences between the groups. Paroxysmal AF was more frequent in the pentaspline (61.0%) and balloon-in-basket (60.0%) groups, while the lattice-tip group showed a 35.6% prevalence (*P* = 0.003). Notably, atrial tachycardia was significantly more prevalent in the lattice-tip group (25.4%) compared with the pentaspline (0.0%) and balloon-in-basket (0.0%) group (*P* < 0.001).

**Table 1:** Baseline characteristics.

Patient baseline characteristics	Pentaspline *n* = 107	Balloon-in-basket *n* = 71	Lattice-Tip *n* = 61	*P*-value
Age (years)	69 (63–77)	70 (62–76)	67 (61–76)	0.602
Male Sex (%)	82 (60.3%)	38 (51.1%)	44 (72.1%)	**0.049**
Paroxysmal AF (%)	65 (61.0%)	42 (60.0%)	21 (35.6%)	**0**.**003**
Persistent AF (%)	41 (38.7%)	28 (40.0%)	23 (39.0%)	0.987
Atrial Tachycardia (%)	0 (0.0%)	0 (0.0%)	15 (25.4%)	**<0**.**001**
BMI (kg/m^2^)	26 (23–31)	27 (25–31)	27 (24–29)	0.310
Left atrial diameter (mm)	43 (37–48)	45 (39–49)	43 (40–47)	0.694
EF (%)	60 (60–60)	60 (59–60)	60 (55–65)	0.836
Baseline GFR	80 (65–110)	78 (64–108)	86 (64–108)	0.816
Hypertension (%)	73 (68.9%)	51 (71.8%)	36 (61.1%)	0.401
Diabetes mellitus (%)	17 (16.0%)	9 (12.7%)	4 (6.8%)	0.231
History of stroke or TIA (%)	7 (6.6%)	2 (2.8%)	6 (10.2%)	0.367
Coronary artery disease (%)	17 (16.0%)	12 (16.9%)	3 (5.1%)	0.067
Heart failure (%)	13 (12.3%)	8 (11.3%)	6 (10.2%)	0.920
Failed AAD (%)	54 (50.5%)	55 (77.5%)	48 (78.6%)	**< 0.001**

Abbreviations: AAD, anti-arrhythmic drug; AF, atrial fibrillation; BMI, body mass index; CAD, coronary artery disease; EF, ejection fraction; GFR, glomerular filtration rate; IQR, interquartile range; TIA, transient ischaemic attack.

The median procedural time was 35 min (28–55) for the pentaspline catheter, 45 min (40–60) for the balloon-in-basket, and 60 min (50–80) for the lattice-tip catheter. Regarding energy delivery, the median number of pulsed field (PF) applications was 40 (40–40) for the pentaspline, 24 (24–24) for the balloon-in-basket, and 39 (20–60) for the lattice-tip catheter. RF applications were utilized only for the lattice- tip, with a median of 16 (7–23). Substrate modification was performed exclusively with the lattice-tip catheter. In the lattice-tip group, the most frequently performed lesion sets were posterior wall isolation (45%) and anterior line ablation (41%), followed by repeat PVI (36%), cavotricuspid isthmus (CTI) ablation (30%), and roof line ablation (25%). Detailed procedural data are summarized in *Table 2*.

**Table 2:** Baseline characteristics, continued.

Procedural data	Pentaspline *n* = 107	Balloon-in-basket *n* = 71	Lattice-tip *n* = 61
Procedural time (min)	35 (28–55)	45 (40–60)	60 (50–80)
Fluoroscopy time (min)	8.2 (5.5–12.6)	7.2 (5.5–11.5)	6.3 (4.5–8–4)
Fluoroscopy dose (µGym^2^)	219.99 (112.9–357.3)	291.1 (153.8–723)	232 (132.48–507.07)
Anterior Line (%)	—	—	25 (41)
Roof Line (%)	—	—	15 (25)
Posterior wall (%)	—	—	27 (45)
CTI Ablation (%)	—	—	18 (30)
Re-PVI	—	—	22 (36)
Number of PF applications (IQR)	40 (40–40)	24 (24–24)	39 (20–60)
Number of RF applications (IQR)	—	—	16 (7–23)
Total Fluid Adminstration (mL)	427 (275–537)	525 (431 -544)	726 (512–1003)

Total fluid administration differed significantly among catheter technologies, being highest in the lattice-tip group 726 mL (512–1003), followed by the balloon-in-basket 525 mL (431–544) and pentaspline groups 427 mL (275–537). Pairwise comparisons demonstrated significant differences between all catheter systems (pentaspline vs. balloon-in-basket: adj. *P* = 0.016; pentaspline vs. lattice-tip: adj. *P* < 0.001; balloon-in-basket vs. lattice-tip: adj. *P* = 0.001).

Renal function at baseline, assessed by Cockcroft–Gault estimated glomerular filtration rate and creatinine value, did not differ significantly across groups (*P* = 0.816 and *P* = 0.144 respectively). Baseline laboratory values before and after the procedure are summarized in *Tables 3* and *4*.

**Table 3:** Baseline laboratory markers stratified by catheter type. Data are expressed as median (interquartile range).

Marker	Pentaspline	Balloon-in-basket	Lattice-tip	*P*-value
Haemoglobin (g/dL)	13.0 (11.8–14.4)	13.0 (12.0–13.9)	13.6 (12.6–15.3)	**0**.**020**
LDH (U/L)	190 (167–218)	206 (185–227)	202 (174–226)	0.087
Total Bilirubin (mg/dL)	0.70 (0.50–0.82)	0.60 (0.40–0.82)	0.60 (0.50–0.90)	0.250
Indirect Bilirubin (mg/dL)	0.32 (0.25–0.52)	0.31 (0.20–0.48)	0.38 (0.25–0.62)	0.070
Haptoglobin (mg/dL)	99.8 (70.2–122)	108 (82.7–137)	95.8 (75.7–123)	0.184
Serum creatinine (mg/dL)	0.92 (0.76–1.06)	0.93 (0.75–1.07)	0.97 (0.81–1.15)	0.144
Troponin (ng/L)	16.0 (9–21)	15 (9–22)	12 (8–15)	0.054
CK (U/L)	83 (60–125)	87 (62–139)	90 (56–135)	0.991
AST (U/L)	24(20–29)	23 (20–30)	23 (20–31)	0.776

**Table 4:** Comparison between pre-procedural and post-procedural median values for each marker, categorized according to the specific catheter technology used.

Marker	Pentaspline			Balloon-in-basket			Lattice-tip		
	Pre	Post	*P*-value	Pre	Post	*P*-value	Pre	Post	*P*-value
Haemoglobin (g/dL)	13.0	12.8	0.672	13.0	13.0	0.995	13.6	12.8	<0.001
LDH (U/L)	190	264	<0.001	206	248	<0.001	202	201	0.017
Total Bilirubin (mg/dL)	0.7	1.0	<0.001	0.6	0.8	<0.001	0.6	0.9	<0.001
Indirect Bilirubin (mg/dL)	0.32	0.61	<0.001	0.31	0.46	<0.001	0.38	0.52	0.004
Haptoglobin (mg/dL)	99.8	55.7	<0.001	108.0	99.2	0.201	95.8	96.7	0.073
Serum creatinine (mg/dL)	0.92	0.95	0.002	0.93	0.88	0.576	0.97	0.95	0.010
Troponin (ng/L)	16	1272	<0.001	15	1476	<0.001	12	757	<0.001
CK (U/L)	83	286	<0.001	87	311	<0.001	90	162	<0.001
AST/GOT (U/L)	24	58	<0.001	23	59	<0.001	23	42	<0.001

### Change in haemolysis markers

**Figure 1:**
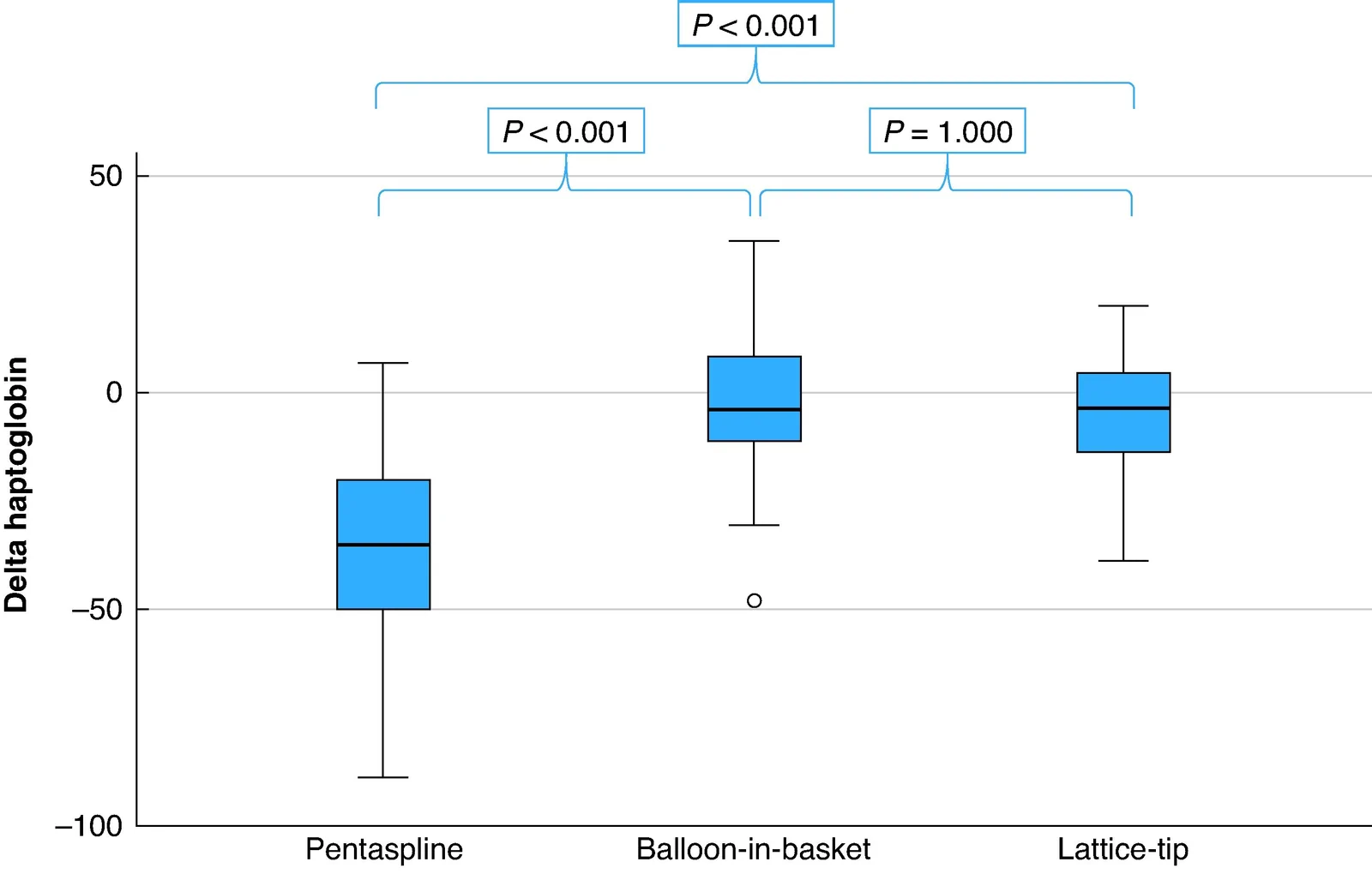
Comparison of the median change in haptoglobin nΔ haptoglobin among the three catheter designs. Data are all presented as medians with interquartile ranges (IQR).

In patients treated with the pentaspline catheter, a significant reduction in median haptoglobin levels was observed after the procedure compared with baseline [pre 99.8 mg/dL (70.2–122) vs. post 55.7 mg/dL (23.2–91.8); *P* < 0.001]. In contrast, no significant changes in haptoglobin levels were detected with the balloon-in-basket catheter [pre 108 mg/dL (82.7–137) vs. post 99.2 mg/dL (83–141); *P* = 0.201] or the lattice-tip catheter [pre 95.8 mg/dL (74.7–123) vs. post 96.7 mg/dL (79.4–126); *P* = 0.073] (*Table 4* and *Figure 1*).

When comparing the different catheters types, the levels of haptoglobin changes differed significantly (*P* < 0.001; *Table 5*). The most pronounced haptoglobin depletion occurred in the pentaspline group [Δ haptoglobin −35 mg/dL (−50 to −20)], followed by the balloon-in-basket [−4.0 mg/dL (−12.40–9.10)] and lattice-tip catheters [−3.6 mg/dL (−17–3.2)]

**Table 5:** Changes (delta) between pre- and post-ablation values for each marker.

Marker	Pentaspline (Δ)	Balloon-in-basket (Δ)	Lattice-tip (Δ)	*P*-value
Haemoglobin (g/dL)	−0.0	0.1	−0.8	<0.001
LDH (U/L)	70	46	5	<0.001
Total Bilirubin (mg/dL)	0.5	0.2	0.3	<0.001
Indirect Bilirubin (mg/dL)	0.31	0.13	0.15	<0.001
Haptoglobin (mg/dL)	−35	−4.0	−3.6	<0.001
Serum creatinine (mg/dL)	0.04	0.01	−0.06	<0.001
Troponin (ng/L)	1252	1399	742	<0.001
CK (U/L)	198	184	68	<0.001
AST/GOT (U/L)	31	33	16	<0.001

Pairwise post-hoc comparisons confirmed a significant difference between pentaspline and lattice-tip (adj. *P* < 0.001), as well as between pentaspline and balloon-in-basket (adj. *P* < 0.001), whereas no significant difference was observed between the lattice-tip and balloon-in-basket catheters (adj. *P* = 1.00) (*Figure 1*).

**Figure 2:**
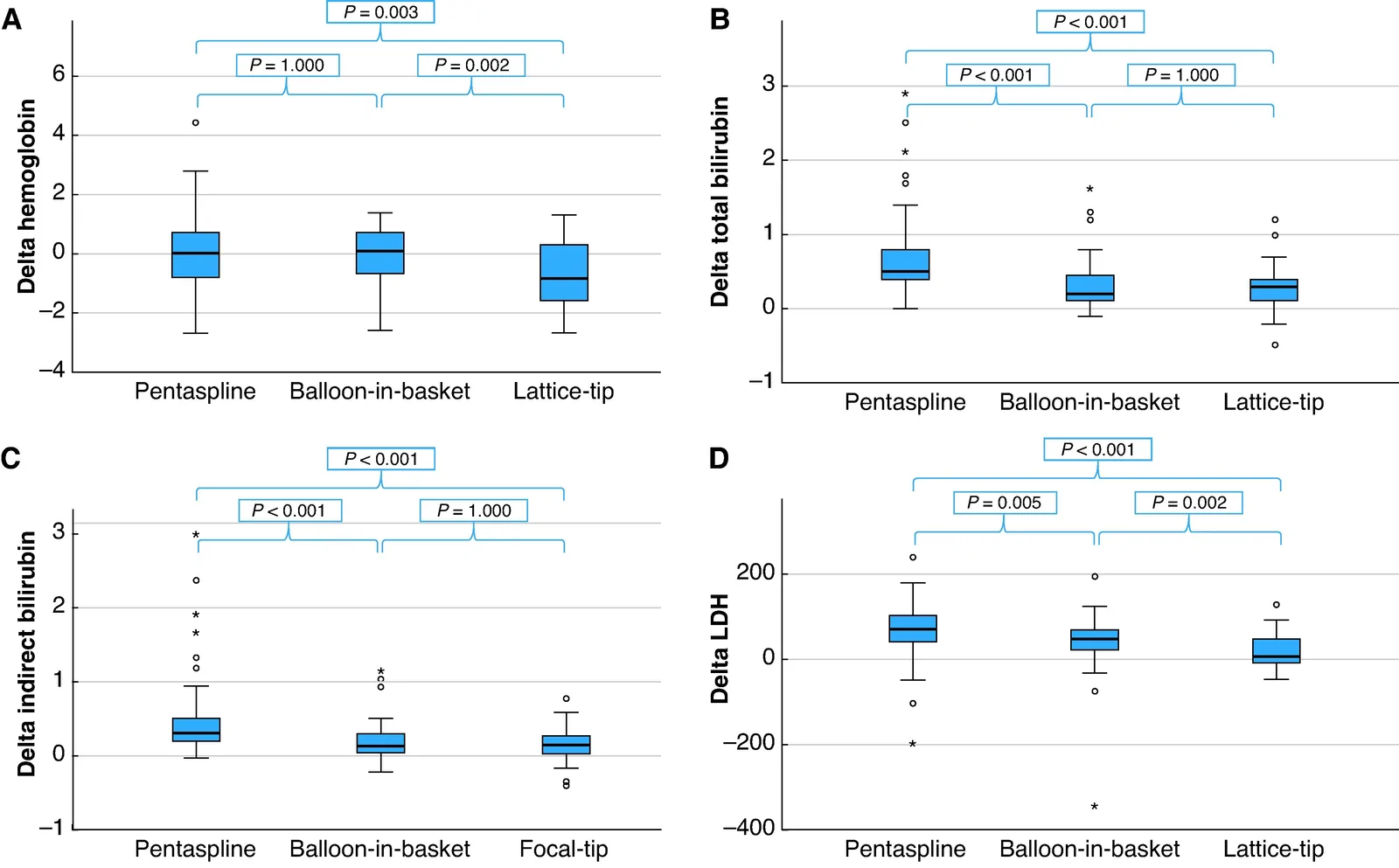
Comparison of the median change (δ) in haemoglobin (panel *A*), total bilirubin (panel *B*), indirect bilirubin (panel *C*), and LDH (panel *D*) among the three catheter configurations. Data are presented as medians with interquartile ranges (IQR). Statistical significance (*P* < 0.05) between different catheter designs is indicated by the respective *P*-values.

LDH levels showed significant post-procedural change for pentaspline [pre 190 U/L (167–218), post 264 U/L (229–288); *P* < 0.001], balloon-in-basket [pre 206 U/L (185–227); post 248 U/L (220–277); *P* < 0.001] and lattice-tip [pre 202 U/L (174–226), post median 201 U/L (188–231); *P* = 0.017] catheters (*Figure 2B*). Periprocedural changes in LDH differed significantly across catheter types (*P* < 0.001) (*Table 5*). The greatest median increase in LDH levels were observed with pentaspline [ΔLDH 70 U/L (38–102)] followed by balloon-in-basket [ΔLDH 46 U/L (21 – −70)] and lattice-tip [ΔLDH 5 U/L (−11–43)]. The pairwise comparison showed a significant difference between pentaspline and balloon-in-basket catheters (adj. *P* = 0.005), pentaspline and lattice-tip catheters (adj *P* < 0.001) and lattice-tip and balloon-in-basket catheters (adj *P* = 0.002) (*Figure 2, B*).

Total bilirubin levels increased significantly after the procedure across all catheter types [pre pentaspline 0.7 mg/dL (0.50 –0.80) vs. post-pentaspline 1.05 mg/dL (0.80 –1.60); pre lattice-tip 0.60 mg/dL (0.50–0.90) vs. post lattice-tip 0.90 mg/dL (0.70–1.10); pre balloon-in-basket 0.60 mg/dL (0.40–0.80) vs. post balloon-in-basket 0.80 mg/dL (0.50–1.10); *P* = <0.001 for all three catheters; *Figure 2C*], as well as indirect bilirubin levels which also showed a consistent post-procedural rise in all groups [pre pentaspline 0.32 mg/dL (0.25–0.52), post-pentaspline 0.61 mg/dL (0.46–0.92), *P* < 0.001; pre lattice-tip 0.38 mg/dL (0.25–0.62), post lattice-tip 0.52 mg/dL (0.37–0.66); *P* = 0.004; pre balloon-in-basket 0.31 mg/dL (0.20–0.48), post balloon-in-basket 0.46 (0.29–0.65); *P* < 0.001]. When comparing the delta values of pre-post procedural changes among catheter types, significant differences were observed for both, total and indirect bilirubin (all *P* < 0.001) (*Table 5*). Greater peri-procedural increase in total bilirubin was associated with the pentaspline catheter [Δtotal bilirubin 0.5 mg/dL (0.4–0.8)] followed by lattice-tip catheter [Δtotal bilirubin 0.3 mg/dL (0.1–0.4)] and balloon-in-basket catheter [Δtotal bilirubin 0.2 mg/dL (0.1–0.5)]. Pairwise comparisons of bilirubin levels showed significant differences between pentaspline and lattice-tip systems (adj. *P* < 0.001) and pentaspline vs. balloon-in-basket (adj. *P* < 0.001), but no significant difference was observed in between lattice-tip and balloon-in-basket systems (adj. *P* = 1.000) (*Figure 2, C*). Similar behaviour was seen in the levels changes of indirect bilirubin with the greatest increased seen with pentaspline [Δindirect bilirubin 0.31 mg/dL (0.19–0.52)] followed by lattice-tip [Δindirect bilirubin 0.15 mg/dL (0.04–0.27)] and balloon-in-basket [Δindirect bilirubin 0.13 mg/dL (0.04–0.30)]. When comparing among systems, significant difference was detected between pentaspline and lattice-tip (adj. *P* < 0.001) and between pentaspline and balloon-in-basket (adj. *P* < 0.001), no significant difference was observed between lattice-tip and balloon-in-basket (adj. *P* = 1.000) (*Table 5*).

### Creatinine change

**Figure 3:**
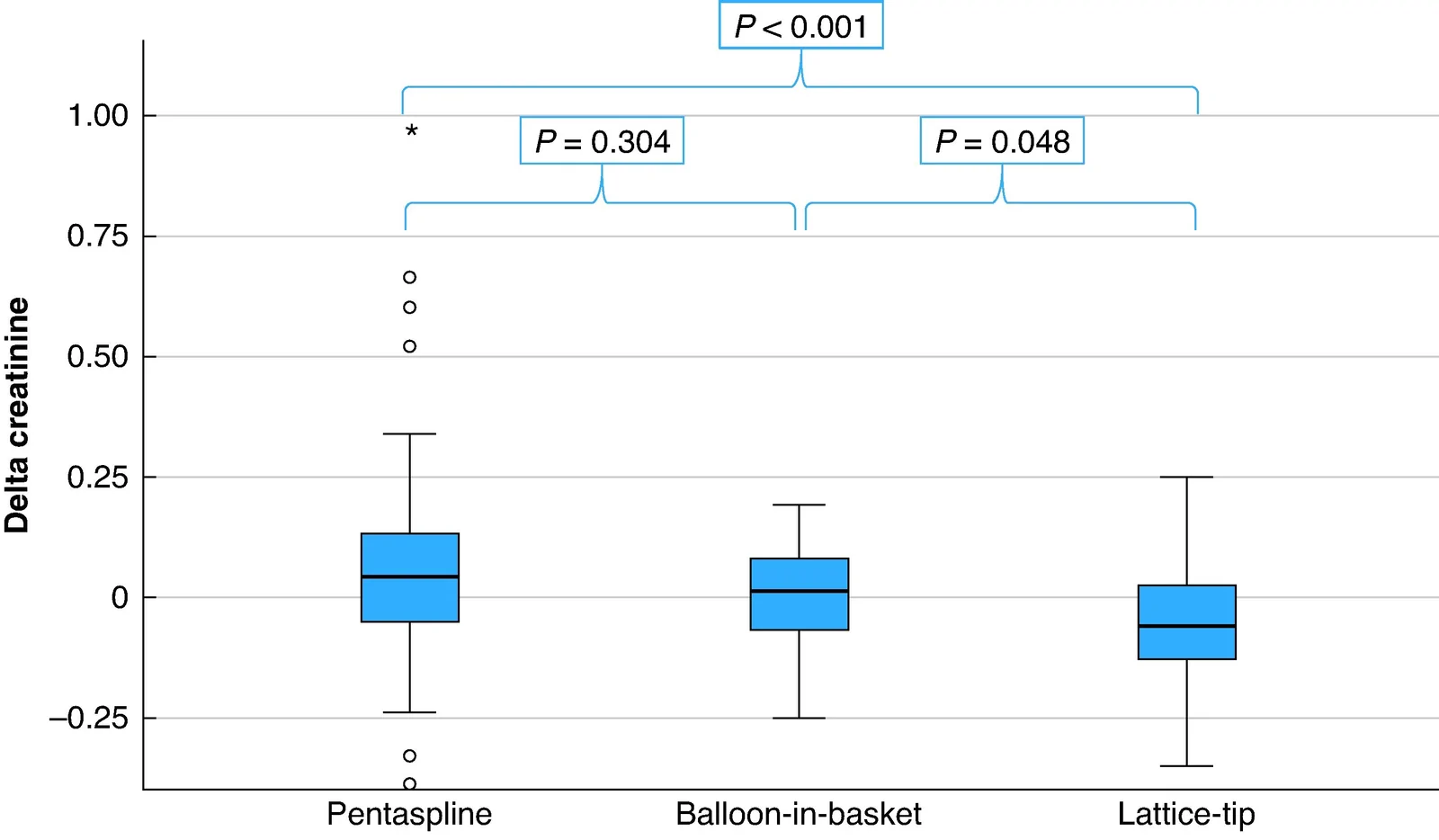
Comparison of the median change in creatinine (δ creatinine) among the three catheter designs. Data are all presented as medians with interquartile ranges (IQR).

When stratified by catheter type, a significant reduction in creatinine levels was detected in patients treated with the lattice-tip catheter (pre 0.97 mg/dL (0.81–1.15), post 0.95 mg/dL (0.85–1.08); *P* = 0.010), and a significant increase was observed in the pentaspline patients (pre 0.92 mg/dL (0.76–1.06), post 0.95 mg/dL (0.84–1.06); *P* = 0.002) whereas no significant change in the balloon-in-basket group (pre 0.93 mg/dL (0.75–1.07), post 0.88 mg/dL (0.79–1.05); *P* = 0.576). Pre and post-procedural changes in serum creatinine levels according to catheter type are shown in *Table 4* and *Figure 3*.

Comparison of the levels of creatinine changes among catheter types demonstrated a significant overall difference (*P* < 0.001; *Table 5*). Greatest increased was observed in the pentaspline group [Δ crea 0.04 mg/dL (−0.05–0.13)] followed by the balloon-in-basket group [Δcrea 0.01 mg/dL (−0.08–0.09)]; contrarily a creatinine decrease was registered in the lattice-tip group [Δcrea −0.06 mg/dL (−0.13–0.05)]. Post-hoc pairwise analyses revealed that changes in creatinine were significantly different between the lattice-tip and pentaspline groups (adj. *P* < 0.001), no significant difference was observed between pentaspline and balloon-in-basket (adj. *P* = 0.304) borderline significant difference was observed between lattice-tip and balloon-in-basket groups (adj. *P* = 0.048) (*Figure 3*).

**Figure 4:**
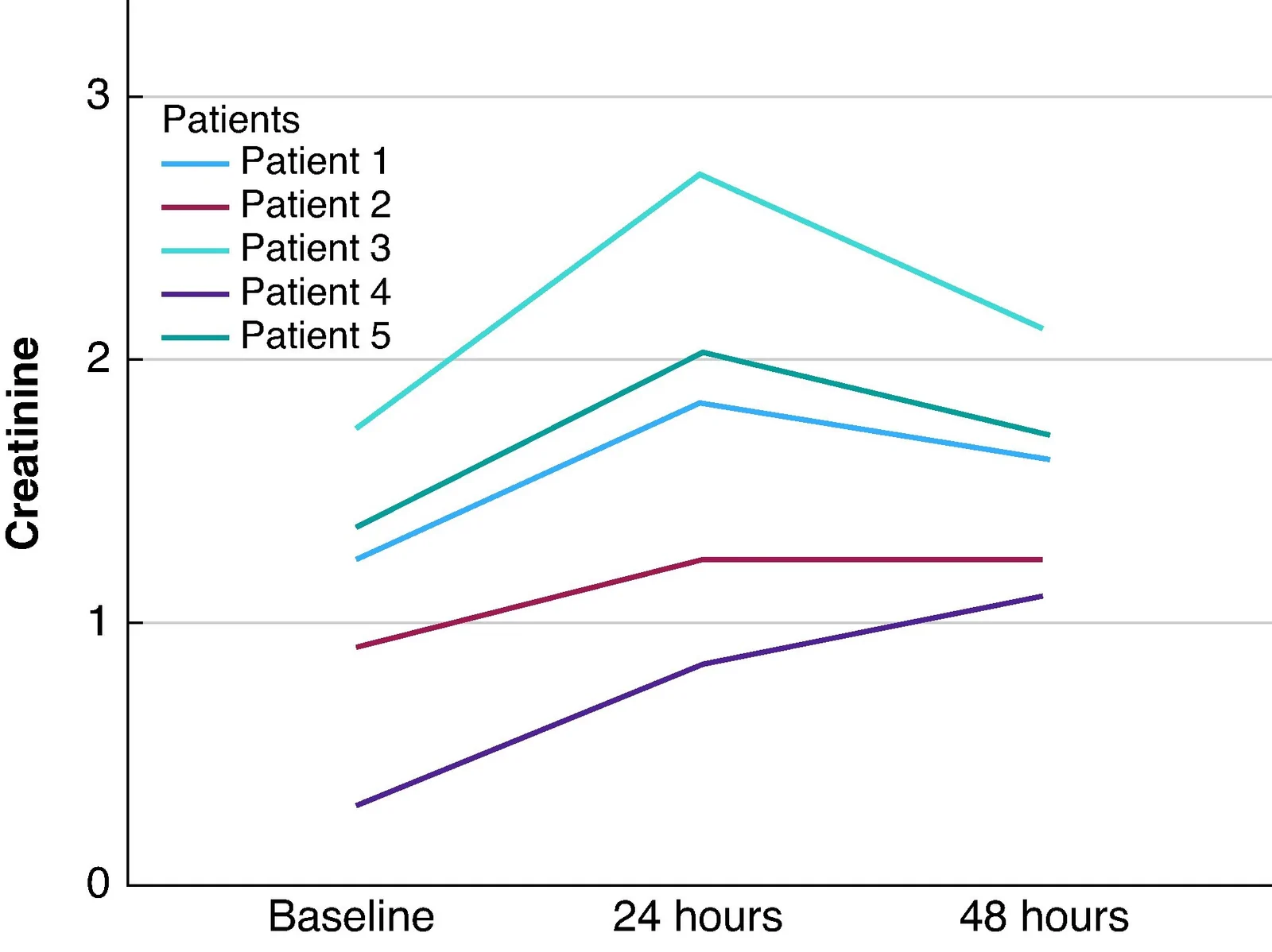
Individual patient trajectories for serum creatinine measured at baseline, pre-procedure, post-procedure day 1, and post-procedure day 2.

Guideline-defined AKI was exclusively observed in the pentaspline group for a total of 5 patients [Δ crea 0.60 mg/dL (0.430–0.81), all KDIGO stage I], leading to a significant difference among the three catheters (pentaspline: 4.9% vs. balloon-in-basket: 0.0% and lattice-tip: 0.0%; *P* = 0.020). Management consisted of additional intravenous fluid administration in three of the five patients. No patient required nephrology consultation, renal replacement therapy, or any other specific intervention. At repeat testing the following day, creatinine levels remained stable or showed a downward trend (*Figure 4*).

### Myocardial damage

**Figure 5:**
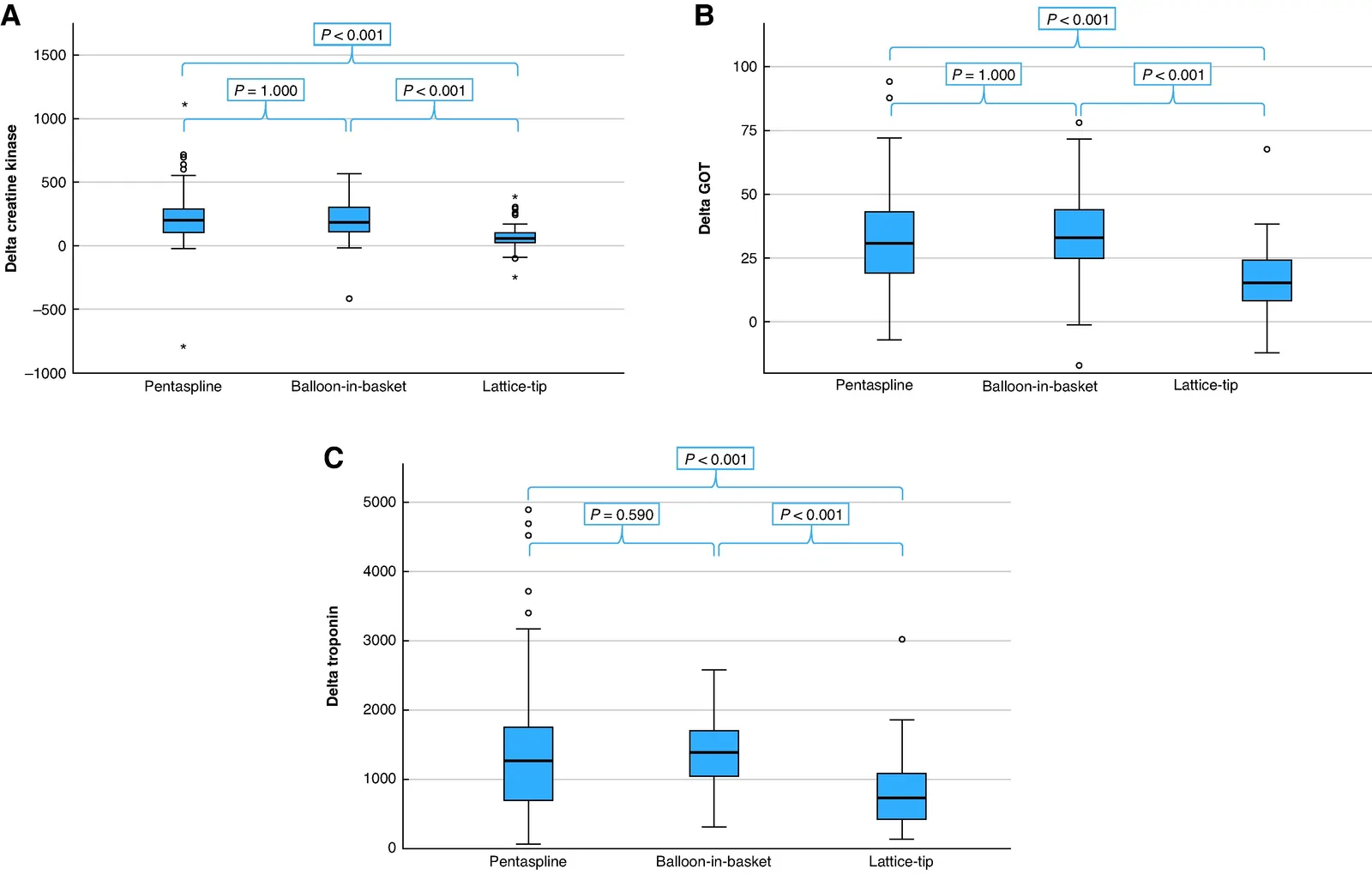
Comparison of the median change (δ) in CK-MB (panel *A*), GOT/AST (panel *B*), and troponin (panel *C*) among the three catheter configurations. Data are presented as medians with interquartile ranges (IQR). Statistical significance (*P* < 0.05) between different catheter designs is indicated by the respective *P*-values.

A significant post-procedural increase in myocardial biomarkers was observed across all catheter types, consistent with procedural-related myocardial injury. The pentaspline group presented significant increase in all three biomarkers levels [TnT: pre 16 ng/L (9–21), post 1272 ng/L (741–1726). CK: pre 83 U/L (60–125), post 311 U/L (240–427). AST: pre 24 U/L (20–29), post 58 U/L (45–69); all *P* < 0.001], similarly for the balloon-in-basket [TnT: pre 15 ng/L (9–22), post 1476 ng/L (1091–1901). CK: pre 87 U/L (62–139), post 311 U/L (240–427). AST: pre 23 U/L (20–30), post 59 U/L (48–74); all *P* < 0.001] and the lattice-tip group [TnT: pre 12 ng/L (8–15), post 757 ng/L (427–1113). CK: pre 90 U/L (56–135), post 162 U/L (112–235). AST: pre 23 U/L (20–31), post 42 U/L (34–52); all *P* < 0.001]. Pre- and post-procedural changes in myocardial injury markers, including high-sensitivity TnT, CK, and GOT, are reported in *Table 4* and *Figure 5*.

When comparing the level of biomarker changes among catheter types, significant differences were observed for troponin T, CK, and GOT (*P* < 0.001). The greatest absolute increase for all three markers was observed in the pentaspline group [ΔTnT 1252 ng/L (705–1760), ΔCK 198 U/L (103–298), ΔGOT 31 U/L (19–43)] followed by the balloon-in-basket group [ΔTnT 1399 (1030–1713), ΔCK 184 U/L (106–309), ΔGOT 33 U/L (24–45)] and the lattice-tip group [ΔTnT 742 ng/L (414–1080), ΔCK 68 U/L (24–111), ΔGOT 16 U/L (7–24)].

Post-hoc pairwise analyses demonstrated larger post-procedural increases in myocardial injury markers in patients treated with the pentaspline and catheter compared with the lattice-tip systems (adj. *P* < 0.001 for all three markers). Similar results were observed when comparing the balloon-in-basket with the lattice-tip catheter (adj. *P* < 0.001 for all three markers) No differences were observed between the pentaspline and balloon-in-basket catheters across myocardial injury endpoints (adj. *P* = 0.590 for TnT; *P* = 1.000 for CK and GOT/AST) (*Figure 4*).

## Discussion

In this large cohort, we systematically evaluated periprocedural biochemical changes associated with different PFA catheter technologies. By comparing markers of haemolysis, renal function, and myocardial injury across three contemporary ablation systems, including the recently introduced balloon-in-basket catheter, our analysis provides a comprehensive assessment of the biological footprint of current PFA technologies in real-world clinical practice.

Our main findings are:

1. The extent of haemolysis differed significantly across contemporary PFA catheter platforms, with the pentaspline catheter showing the most pronounced reduction in haptoglobin and the largest increases in complementary haemolysis markers.
2. These haemolysis-related changes translated into a modest but significant transient increase in serum creatinine exclusively in the pentaspline group, with guideline-defined AKI occurring only in this group.
3. Despite their similarly large-footprint single-shot design, the balloon-in-basket system demonstrated substantially less haemolysis than the pentaspline catheter, suggesting that catheter architecture and tissue-contact assessment mechanisms may importantly modulate blood-pool energy exposure.
4. Myocardial injury was observed with all three PFA systems, but was significantly greater with the pentaspline and balloon-in-basket catheters than with the lattice-tip system, which may indicates relevant differences in lesion footprint and energy delivery characteristics across platforms.

### Haemolysis

Comparative data across currently available PFA catheter platforms remain limited. In this context, our study provides novel insights into the distinct biological footprints associated with contemporary PFA catheter designs. Importantly, baseline characteristics were largely balanced across catheter groups, supporting the robustness of the observed biomarker differences and aligning well with patient profiles reported in major contemporary registries.^4,11^

A significant reduction in haptoglobin levels was observed exclusively in patients treated with the pentaspline catheter, whereas no significant decrease occurred with the lattice-tip or balloon-in-basket systems. This was confirmed by comparing periprocedural haptoglobin changes across catheter types, demonstrating significantly greater haptoglobin-related changes with the pentaspline catheter compared with the lattice-tip and balloon-in-basket systems, while no significant differences were observed between the lattice-tip and balloon-in-basket catheters. Other haemolysis markers, including total and direct bilirubin, showed concordant patterns, supporting the robustness of this endpoint.

*Several mechanisms may plausibly contribute to the greater haemolytic burden observed with the pentaspline system. The larger geometric footprint and increased total electrode surface area of this multi-spline design generates a more extensive interface with the surrounding blood pool, which may be one of the main contributors of the effect on haemolysis observed in our study. This is particularly evident in the basket configuration, where it leads to greater energy dissipation into the circulating blood*.^21^ It is also worth mentioning that no 3D mapping was used when ablating with the pentaspline system, and contact was assessed purely radiographically.

Some retrospective studies have compared haemolysis risk among different PFA catheter designs. Despite some heterogeneity in study design, these investigations consistently suggest that the Affera Sphere-9 system is associated with smaller increases in haemolysis markers compared with larger, multi-electrode configurations (Farawave, Boston Scientific; PulseSelect, Medtronic and Varipulse, Biosense Webster),^21,22^ a finding which is consistent with our data. A recent comparative analysis reported similar findings, identifying the lattice-tip system as the least haemolytic PFA technology when compared with single-shot catheter designs, while reporting similarly low rates of AKI across technologies.^23^ This may be attributable to the smaller total electrode surface area and more localized energy delivery of the Sphere-9 compared with the larger footprints of ‘basket’ or ‘flower’ shaped catheters.

Furthermore, in contrast to the pentaspline catheter, impedance-based contact assessment and real-time temperature rise during energy delivery provide additional confirmation of effective tissue contact.

Notably, the balloon-in-basket system showed a less pronounced degree of haemolysis, consistent with prior reports^24^ and preclinical studies as well,^25^ despite comparable myocardial injury relative to the pentaspline catheter, with both system employing a large-footprint single-shot design. This observation may be explained by the use of impedance-based sensing, which enables a more accurate assessment of tissue contact and thereby facilitates preferential energy delivery to the myocardium rather than dissipation into the blood pool and the insulating effect of the balloon, which may reduce direct exposure of the splines to the circulating blood pool. In addition, our protocol incorporates routine angiography prior to energy delivery to assess pulmonary vein occlusion, providing an additional level of confirmation of adequate catheter–tissue contact and potentially further minimizing inadvertent energy exposure to circulating blood cells. These observations are particularly relevant given the ongoing debate on PFA as a potential standard ablation technology, as haemolysis has emerged as a unique PFA-related limitation compared with thermal energy sources. Particular attention may be warranted in patients with pre-existing chronic kidney disease and reduced renal reserve. In such settings, the cumulative haemolytic burden may be of greater clinical relevance and may warrant careful consideration of the ablation technology used.^6^ However, our findings suggest that the magnitude of this phenomenon is strongly influenced by catheter design and remains largely subclinical in contemporary practice.

### Renal function and acute kidney injury

Although statistically significant changes in serum creatinine were observed across catheter groups, their direction differed according to catheter type. A modest but significant increase in creatinine levels was only observed in the pentaspline group, possibly reflecting the greater degree of haemolysis observed with this catheter. Although an AKI (according to the KDIGO definition of AKI) was observed in five patients [Δ crea 0.60 mg/dL (0.430–0.81), all KDIGO stage I] treated with the pentaspline catheter. By the following day, creatinine levels remained stable or showed a downward trend, with no evidence of further deterioration in renal function. Supportive management consisted of increased intravenous fluid administration in three out of five patients, while no patient required nephrology consultation, renal replacement therapy, or other specific interventions. In contrast, a significant decrease was observed in patients treated with the lattice-tip, a finding likely related to procedural factors such as longer procedure duration and higher intravenous fluid administration. This hypothesis is further supported by the analysis of periprocedural fluid administration, which demonstrated significant differences among catheter groups. The lattice-tip group received the highest volume of fluids [726 mL (512–1003)]—likely due to both catheter irrigation and longer procedure times—followed by the balloon-in-basket [525 mL (431–455)] and pentaspline groups [427 mL (275–537)] (*P* < 0.001). Importantly, no clinically relevant renal events were observed during follow-up in the entire cohort, irrespective of the catheter used. The study first describing PFA-associated AKI, a threshold of more than 70 applications was found to have the highest sensitivity and specificity for predicting haemolysis. Notably, the two index patients who developed AKI in that cohort received 174 and 126 applications, respectively.^12^ In one study, patients who developed severe AKI (serum creatinine >2.5 mg/dL) received a mean of 95 applications compared with 47 applications in those with haemoglobinuria but less severe renal injury.^26^ However, in the large MANIFEST-US registry of over 41 000 patients, the 7 patients requiring dialysis had a mean of only 67 applications (range 54–91), though most had other complicating factors.^11^ Another recent study indicated that the highest degrees of haptoglobin reduction and haemolytic severity are observed when exceeding a threshold of 54 applications.^16^ These figures stand in stark contrast to our limited-application approach, with the median number of applications in our cohort remaining substantially lower than these reported thresholds, underscoring the safety of our protocol and likely explaining why the biological responses observed in our cohort remained subclinical and transient, and also highlighting how a limited-application approach is key to mitigating the risk of renal injury. Additionally, no increase in creatinine was observed with the balloon-in-basket system, most likely attributable to the aforementioned factors, including improved tissue contact and the consequently less pronounced haemolysis.

Our findings suggest that these PFA technologies can be employed safely, provided that the number of applications is carefully managed. By adhering to the ‘olive protocol’ for the pentaspline catheter (10 applications per vein, median 40 applications in our cohort)^19^ and to the ‘pear protocol’ for the balloon-in-basket system (6 applications per vein, median 24 total applications), the cumulative energy delivery remains below the levels associated with severe haemolytic complications in previous studies,^12^ even though both protocols employ a greater number of applications than the manufacturer-recommended protocol used in the device’s pivotal studies.^10,24^ However, it is also important to consider that the ‘olive’ configuration may be delivered with limited tissue contact, potentially resulting in greater energy exposure to the blood pool and contributing to haemolysis, even within a limited-application protocol.

It is finally worth mentioning that prophylactic hydration may mitigate the renal impact of PFA-related haemolysis,^27^ while high-flow catheter irrigation may further reduce haemolytic burden and represents a promising area for future procedural optimization.^28^

### Myocardial injury and lesion footprint

Myocardial injury markers, specifically troponin and AST/GOT, increased significantly following PFA across all three catheter groups, reflecting the expected biological response to myocardial ablation. However, the magnitude of biomarker release varied substantially according to catheter configuration. Our analysis revealed that while all platforms induced myocardial injury, the increase was significantly more pronounced in both the pentaspline and balloon-in-basket groups compared with the lattice-tip catheter. Notably, no statistically significant difference in biomarker elevation was observed between the pentaspline and balloon-in-basket systems. This discrepancy in the scale of myocardial injury may be attributed to the larger footprint area of the pentaspline and balloon-in-basket designs, which deliver energy over a broader tissue surface compared with the more localized delivery of the lattice-tip system. It is important also to acknowledge that comparisons of myocardial injury biomarkers between the lattice-tip group and the pentaspline/balloon-in-basket groups should be interpreted with caution. Unlike the latter systems, which were used exclusively for PVI, the lattice-tip catheter was frequently employed to create additional linear lesion sets. Consequently, differences in the extent and distribution of ablated myocardium may have influenced biomarker release.

LDH, traditionally utilized as a haemolytic marker, exhibited a distinct trend compared with other biomarkers. Significant differences were observed across the three catheter types, with the most pronounced increase occurring in the pentaspline group, followed by the balloon-in-basket and lattice-tip cohorts. This pattern underscores the dual nature of LDH, which may reflect both intravascular haemolysis and myocardial cytolysis in the setting of PFA.

## Limitations

This study has several limitations. Its observational, single-centre design limits generalizability, and residual confounding cannot be fully excluded despite balanced baseline characteristics. Catheter selection was not randomized and was influenced by arrhythmia substrate and operator preference, potentially affecting procedural complexity and energy delivery. Laboratory measurements were obtained at predefined time points and may not fully capture the temporal dynamics of biomarker release.

A heterogeneous distribution of arrhythmias across groups was observed. Specifically, the balloon-in-basket and pentaspline catheters were utilized exclusively for *de novo* ablations, whereas the lattice-tip catheter was reserved for repeat procedures. This unequal distribution of ablation targets and lesion types (PVI for pentaspline/balloon-in-basket and linear lesions for lattice-tip) among the study groups may have contributed to differences in biomarker responses and should therefore be considered when interpreting comparisons between the three catheter technologies. Furthermore, not only the different lesions set but also the use of RF exclusively in the lattice-tip group may influence the comparison in terms of myocardial biomarkers.

Finally, the standardized, limited-application protocols utilized in this study may themselves represent a limitation regarding the generalizability of our findings. Given the known linear correlation between the number of energy deliveries and haemolytic severity, our relatively low procedural burden may have precluded the observation of more severe clinical or laboratory-based complications. Furthermore, the median number of PFA applications in the lattice-tip group was lower than that typically required for a *de novo* PVI procedure. Consequently, the haemolytic burden observed in this group may not fully reflect that associated with a PVI-only strategy using the same catheter. Consequently, our results specifically reflect the safety profile of our ablation strategy and may not be fully generalizable to centres or clinical scenarios requiring a substantially higher number of PFA applications.

## Conclusion

In this large PFA cohort, catheter design may be one of the main contributor to the extent of haemolysis, renal response, and myocardial injury, with the pentaspline catheter being associated with the greatest haemolytic effect and the highest incidence of transient AKI. In addition, the extent of myocardial injury appears to correlate directly with catheter size, likely reflecting the larger ablation footprint and greater tissue surface contact of larger devices. Overall, contemporary PFA systems appear safe and can be administered without substantial concern for hemodynamically relevant haemolysis or AKI, provided that procedural volume is carefully managed and cumulative energy delivery remains within the safety ranges established in the literature.

## Data Availability

Anonymized data underlying this article will be shared on reasonable request to the corresponding author, in accordance with applicable institutional policies.
